# Expression of Toll-like receptors in the mucosa of patients with ulcerative colitis

**DOI:** 10.3892/etm.2015.2258

**Published:** 2015-02-04

**Authors:** YUJING FAN, BINGRONG LIU

**Affiliations:** Department of Gastroenterology, The Second Affiliated Hospital of Harbin Medical University, Harbin, Heilongjiang 150080, P.R. China

**Keywords:** Toll-like receptor, ulcerative colitis, mucosa

## Abstract

Patients with ulcerative colitis (UC) have a high risk of developing colorectal cancer. The aim of the present study was to evaluate the expression pattern of Toll-like receptors (TLRs) in the colonic mucosa of patients with UC. Colonic mucosal biopsy specimens were collected during colonoscopy from 30 patients with UC and 30 patients with normal findings as controls. The protein and mRNA expression levels of TLRs 1-4 and TLR9 were measured by immunohistochemistry and reverse transcription-quantitative polymerase chain reaction analysis, respectively. The results showed that the mRNA and protein expression of TLR2, TLR4 and TLR9, but not TLR1 and TLR3, was significantly increased in the colonic mucosa of patients with UC compared with that in the normal controls. TLR (TLR2, TLR4 and TLR9) immunoreactivity was found in the cytoplasm of epithelial cells in the mucosa, and occasionally in the endothelium of small vessels of the stromal tissues. In conclusion, TLR2, TLR4 and TLR9 expression may be important in the biological pathogenesis of UC. TLR alterations in the innate response system may contribute to the pathogenesis of UC.

## Introduction

Ulcerative colitis (UC) is a relapsing and remitting disease characterized by acute non-infectious inflammation of the colorectal mucosa. The rectal mucosa is invariably affected. The incidence of UC is between 1.2 and 20.3 per 100,000 individuals per year, and its prevalence is between 7.6 and 246.0 per 100,000 individuals per year (1). Although the etiology of UC has yet to be fully elucidated, it has been suggested that environmental factors, such as gut microbiota, stimulate the inappropriate activation of mucosal immunity, thus leading to inflammation (2). Intestinal homeostasis is dependent on a controlled innate immune response to the microbiota, which are recognized by Toll-like receptors (TLRs) on epithelial and immune cells (3).

TLRs comprise an important family of type I transmembrane receptors that allow immune cells to recognize pathogens and trigger inflammatory responses, and are expressed not only in a variety of immune cells but also in non-immune cells, such as fibroblasts and epithelial cells (4). TLRs are constitutively or inducibly expressed by a number of different cell types in the gastrointestinal tract, including intestinal epithelial cells and monocytes/macrophages, dendritic cells of the lamina propria and myofibroblasts, endothelial cells and adipocytes of the intestinal submucosa. When genetic predisposition or environmental stimuli impair mucosal or commensal homeostasis, certain intestinal diseases, such as Crohn’s disease (CD), may develop (4–6).

Aberrant TLR signaling can induce tissue damage and barrier destruction through the overproduction of cytokines and chemokines and the loss of the commensal-mediated responses of colonic epithelial progenitors (7); however, although previous studies (8–10) have investigated the role of TLRs in inflammatory bowel disease (IBD), the expression of TLRs and their role in microbial recognition in innate immunity have not, to the best of our knowledge, been extensively evaluated in the colonic mucosa of patients with UC. The aim of the present study was to determine the alterations in the expression of the main conductors of the innate immune response, including TLR1, TLR2, TLR3, TLR4 and TLR9, in the colonic mucosa of patients with UC and normal controls. The protein and mRNA levels of TLRs 1–4 and TLR9 were evaluated by immunohistochemical techniques and reverse transcription-quantitative polymerase chain reaction analysis, respectively.

## Materials and methods

### Clinical samples

Following the provision of informed consent, unrelated patients with UC (n=30) were recruited from the Outpatient Clinic at the Department of Gastroenterology of The Second Affiliated Hospital of Harbin Medical University (Harbin, China). The patients all fulfilled the diagnostic requirements of UC according to the Chinese Medical Association criteria. Unrelated healthy individuals with a normal colonoscopy (n=30) were randomly recruited from the same hospital. Two colonic biopsy specimens were collected from each participant for routine histological examination. One sample was fixed in 10% formalin for immunohistochemistry. The other sample was immediately frozen in liquid nitrogen and stored at −80°C for later RNA extraction.

All subjects were of Chinese Han descent, and the patients and controls were matched for age and gender. This study was approved by the Ethical Review Committee of Research in Harbin Medical University.

### Immunohistochemistry

Samples fixed in 10% formalin were subsequently embedded in paraffin, and sections of 4-mm thickness were cut from the formalin-fixed samples. The sectioned tissue was deparaffinized in xylene and then rehydrated in a graded ethyl alcohol series. For increased specificity and sensitivity, tissues were microwaved for 10 min for antigen retrieval. Following cooling and rinsing in distilled water, endogenous peroxide activity was blocked with 3% H_2_O_2_ for 10 min, and the samples were then rinsed in 0.01 mol/l phosphate-buffered saline (PBS, pH 7.4) for 10 min. The sections were subsequently preincubated with a protein blocking solution (ZSGB-BIO, Beijing, China) for 10 min, prior to incubation with the primary polyclonal antibodies against TLR1 (cat. no. sc-30000; rabbit polyclonal), TLR2 (cat. no. sc-10739; rabbit polyclonal), TLR3 (cat. no. sc-32232; mose monoclonal), TLR4 (cat. no. sc-10741; rabbit polyclonal) and TLR9 (cat. no. sc-25468; rabbit polyclonal) (Santa Cruz Biotechnology, Inc., Santa Cruz, CA, USA) at dilutions of 1:300, 1:70, 1:300, 1:100 and 1:50, respectively, at 4°C overnight in a humid chamber. The slides were then washed three times in PBS and incubated with secondary biotinylated antibody (ZSGB-BIO) for 15 min at room temperature. The streptavidin-peroxidase method (ZSGB-BIO)was used to detect the antigen-antibody complexes, and diaminobenzidine (DAB) was used as the chromogen substrate. Peroxidase signals were visualized following 3 min of treatment with a DAB substrate-chromogen system (ZSGB-BIO). Finally, the sections were stained lightly with hematoxylin. For the negative control, PBS was used in place of the primary antibody. All sections were coded and independently examined by two investigators. The staining intensity was scored as negative or weak (−), moderate (+), strong (++) or very strong (+++).

### Reverse transcription-quantitative polymerase chain reaction (RT-qPCR) analysis

The expression rates of the TLR genes were analyzed through RT-qPCR analysis. Total RNA was extracted from fresh frozen tissue using TRIzol™ reagent (Invitrogen Life Technologies, Carlsbad, CA, USA) in accordance with the manufacturer’s instructions. Total RNA (1 μg) was subjected to reverse transcription using a Reverse Transcription system (Promega Biotech Co., Ltd., Beijing, China), according to the manufacturer’s instructions. All primers (Sangon Biotech Co., Ltd., Shanghai, China) used in this study are shown in Table I. For the qPCR, 2 μl cDNA, 1 μl primers and 10 μl qPCR iQ™ SYBR^®^ Green supermix (Bio-Rad, Hercules, CA, USA) was mixed to obtain a final volume of 20 μl. The reaction was followed by 40 cycles at 95°C for 30 sec, 60°C for 30 sec and 72°C for 30 sec. All reactions were performed in duplicate. Amplification of the expected single products was confirmed by dynamic melting curves and by electrophoresis on 1.5% agarose gels stained with ethidium bromide. Fluorescence data were automatically collected and analyzed by iCycler iQ Optical Software (version 3.0a; Bio-Rad). The expression of TLRs 1–4 and TLR9 was examined and normalized to a constitutive gene (GAPDH), and relative induction was calculated using the 2^(−ΔΔCt)^ method (11).

### Statistical analysis

All data are presented as the mean ± standard deviation and were analyzed using SPSS 16.0 statistical software (SPSS, Inc., Chicago, IL, USA). Statistical differences between groups were analyzed with non-parametric methods (Mann-Whitney U-test for unpaired data). The χ^2^ test was used to estimate mRNA values. P<0.05 was considered to indicate a statistically significant difference.

## Results

### Expression of TLR proteins in mucosal biopsies

**Figure 1:**
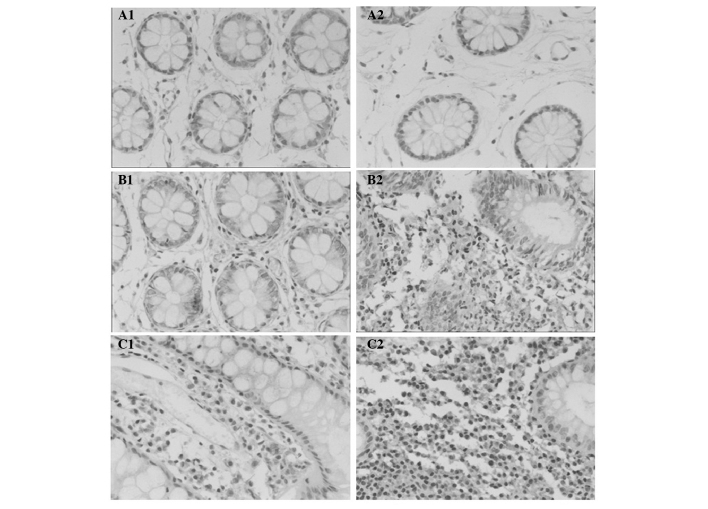
TLR protein immunoreactivity in the mucosa of (A1–C1) control subjects and (A2–C2) patients with UC: (A) TLR2, (B) TLR4 and (C) TLR9. Immunoreactivity was generally observed in the cytoplasm of epithelial cells and ocasionally in the endothelium of small vessels of the stromal tissues. Magnification, ×400. TLR, Toll-like receptor; UC, ulcerative colitis.

Immunohistochemical assessment for TLRs in the mucosal biopsies was performed using anti-TLR antibodies. Representative staining patterns for the TLRs are shown in Fig. 1. Positive staining for TLRs 1–4 and TLR9 were generally observed in the cytoplasm of epithelial cells, and TLR3 was also expressed on the cell membrane. In the stromal non-epithelial tissue, a weak positive reaction was additionally occasionally observed in the endothelium of the small vessels. The expression of TLR2, TLR4 and TLR9 was significantly stronger in the cytoplasm of epithelial cells from the patients with UC than those from the normal controls (Table II). The differences in the expression levels of TLR1 and TLR3 in the colonic mucosa between patients with UC and normal controls were not statistically significant (Table II).

### Levels of TLR mRNA in mucosal biopsies of patients with UC and normal controls

**Figure 2:**
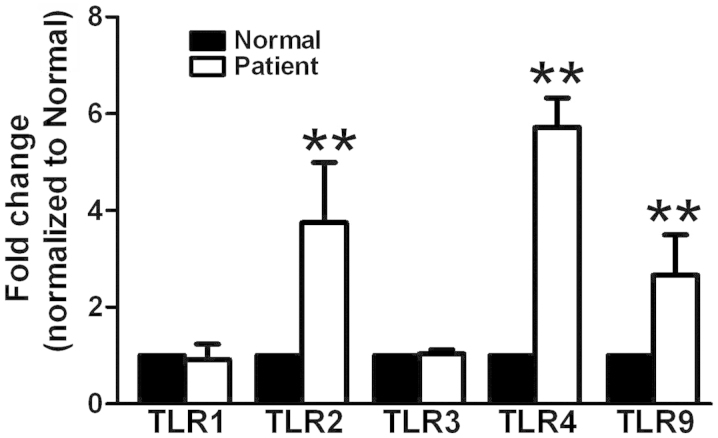
TLR mRNA expression in the mucosal biopsies of controls and patients with UC. ^**^P<0.05, versus controls. TLR, Toll-like receptor.

Similar to the results for protein levels, the mRNA expression levels of TLR2, TLR4 and TLR9, but not TLR1 and TLR3, were significantly higher in the patients with UC than those in the normal controls (Fig. 2).

## Discussion

UC comprises a group of chronic inflammatory disorders involving the mucosa of the colon. Despite a lack of extensive investigation into the role of the adaptive immune response in UC, evidence suggests an association between innate immunity and the pathology of the disease (12). The findings of numerous studies have indicated that the surface epithelium plays a critical role as the front line of the mucosal innate immune system in the gastrointestinal tract (13–15).

The dysregulation of innate and adaptive intestinal immune responses to bacterial microbiota is believed to be the hallmark of IBD pathogenesis. In the present study it has been demonstrated that intestinal epithelial cells constitutively express several functional TLRs, which recognize a variety of distinct microbial components. These TLRs not only play a key role in microbial recognition in innate immunity but also participate in the activation of adaptive immune responses (16). Following the recognition of pathogen-associated molecular patterns by TLRs, signal transduction is initiated. This signaling pathway results in the activation of a number of transcription factors, including activator protein 1, nuclear factor-κB, ETS domain-containing protein Elk-1, cyclic adenosine monophosphate-response element-binding protein and signal transducers and activators of transcription. As a result of the activation of the TLRs, major histocompatibility complex and costimulatory molecules are upregulated and proinflammatory cytokines and chemokines are expressed (17–19).

In the gastrointestinal tract, the polarized expression of TLRs in apical and basolateral compartments has been suggested to be a key factor underlying the discrimination between pathogenic bacteria invading the epithelium and nonpathogenic microbes facing the luminal surface (20–22). TLR ligation on the epithelial cells of the intestine by bacterial products induces epithelial cell proliferation, the secretion of immunoglobulin A into the gut and the expression of antimicrobial peptides (23).

In the present study, it was found that the gene expression of TLR2 and TLR4 was significantly upregulated at the mRNA and protein levels in the inflamed mucosa as compared with the normal controls. By contrast, TLR1 and TLR3 levels were not found to be significantly altered in the patients with UC. These results corroborate those previously reported for TLR2 and TLR4 gene expression in IBD (5,6,17–19). Using immunofluorescence histochemistry, Cario and Podolsky (17) found that TLR3 expression was significantly downregulated in intestinal epithelial cells in active CD but not in UC. By contrast, significant upregulation of TLR4 was found in both UC and CD (17). In a study by Hausmann *et al* (18), the induction of TLR2, TLR4 and TLR5 mRNA was observed in inflammation-stimulated macrophages in the colonic mucosa of patients with IBD.

In the present study it was additionally found that TLR9 gene expression was upregulated in the mucosa of patients with UC as compared with the healthy controls, although in a previous study it was reported that TLR9 expression was downregulated in inflamed colonic mucosa from patients with IBD (24). In normal circumstances, TLR9 combines with unmethylated CpG motifs of the pathogen during bacterial or viral infection, thus stimulating the maturation and activation of dendritic cells and B cells and the elimination of pathogens by the secretion of cytokines and specific antibodies (25). In a study by Hall *et al* (26) it was reported that, in response to commensal bacterial DNA, dendritic cells signaled via TLR9 to inhibit the differentiation of regulatory T cells in the gut. The activation of TLR9 has been shown to result in the maturation of dendritic cells and the release of type 1 T-helper cell cytokines, such as interleukin (IL)-6, IL-12, IL-10 and tumor necrosis factor-α (27–29). Consistent with the present results, previous studies have analyzed TLR9 expression in cells (30,31), and found little TLR9 protein expression in isolated colonic epithelial cells, as assessed by western blot analysis. Gut inflammation with leukocyte infiltration may therefore play a role in the upregulation of TLR9 expression in the colonic mucosa of patients with UC.

In conclusion, the results of the present study have shown overexpression of TLR2, TLR4 and TLR9 in the colonic mucosa of patients with UC, which may be important in the biological pathogenesis of UC. The purpose of this receptor upregulation may be to increase the antigenic stimulation of inflammatory and immune pathways activated by ligand binding. Further studies are necessary to provide an enhanced understanding of the function of these TLRs in the mucosa from patients with UC.

## Figures and Tables

**Table I:** Quantitative polymerase chain reaction primers used for the detection of TLRs.

Gene	Primer sequence (5′-3′)	Amplicon size (bp)
TLR1		102
Sense	AGGTCTTGCTGGTCTTAGGAGA	
Antisense	TGTTTGTGGGGAACACAATGTG	
TLR2		160
Sense	TACTTTGTGGATGGTGTGGGTC	
Antisense	GCTTTTTACAGCTTCTGTGAGC	
TLR3		196
Sense	AACTCAGAAGATTACCAGCCGC	
Antisense	TCAGTCAAATTCGTGCAGAAGG	
TLR4		110
Sense	TCCATTTCAGCTCTGCCTTCAC	
Antisense	ACACCACAACAATCACCTTTCG	
TLR9		154
Sense	CTGCGACCACGCTCCCAACCCC	
Antisense	TCCCAGCCCACGGAACCAACTG	
GAPDH		213
Sense	AAGAAGGTGGTGAAGCAGGC	
Antisense	TCCACCACCCAGTTGCTGTA	

TLR, Toll-like receptor.

**Table II:** TLR protein detection by immunohistochemistry.

	Index of staining pattern					
			
Protein	Negative/weak (−)	Moderate (+)	Strong (++)	Very strong (+++)	Mean rank	P-value
TLR1						0.076
Control	2	9	18	1	34.95	
UC	0	19	11	0	26.95	
TLR2						0.025
Control	4	11	15	0	25.85	
UC	2	9	16	3	35.15	
TLR3						0.205
Control	0	10	18	2	33.03	
UC	2	12	15	1	27.97	
TLR4						0.030
Control	2	12	15	1	26.12	
UC	0	9	19	2	34.88	
TLR9						0.031
Control	6	18	6	0	26.10	
UC	3	13	14	0	34.90	

n=30 per group. TLR, Toll-like receptor; UC, ulcerative colitis.
